# Supplementary material for: Extracellular Vesicles Derived from Opuntia ficus-indica Fruit (OFI-EVs) Speed Up the Normal Wound Healing Processes by Modulating Cellular Responses
Source: Int J Mol Sci. 2024 Jun 28;25(13):7103. doi: 10.3390/ijms25137103 (PMC11241772; doi:10.3390/ijms25137103)
Supplement: Supplementary file 1 [file ijms-25-07103-s001.zip › ijms-3039966-supplementary.pdf]

*Article*

# Extracellular vesicles derived from *Opuntia ficus-indica* fruit (OFI-EVs) speed up the normal wound healing processes by modulating cellular responses

**Anna Valentino [1,2, †,*], Raffaele Conte [1,2, †], Dalila Bousta [3], Hicham Bekkari [4], Anna Di Salle [1,2], Anna Calarco [1,2,5,*] and Gianfranco Peluso [1,2,5]**

[1] Research Institute on Terrestrial Ecosystems (IRET), CNR, Via Pietro Castellino 111, 80131 Naples, Italy. anna.valentino@cnr.it; raffaele-conte@cnr.it; anna.calarco@cnr.it;

[2] National Biodiversity Future Center (NBFC), 90133 Palermo, Italy

[3] National Agency of Medicinal and Aromatic Plants Tounate, Morocco. boustadalila@gmail.com

[4] Laboratory of Biotechnology, environment, agrofood and health (LBEAS). hichambekkari@yahoo.fr

[5] Faculty of Medicine and Surgery, Saint Camillus International University of Health Sciences, Via di Sant'Alessandro 8, 00131 Rome, Italy. gianfranco.peluso@unicamillus.org

† These authors contributed equally to this work

*Correspondence: anna.valentino@cnr.it; anna.calarco@cnr.it

**Citation:** To be added by editorial staff during production.

Academic Editor: Firstname Lastname

Received: date
Revised: date
Accepted: date
Published: date

**Copyright:** © 2024 by the authors. Submitted for possible open access publication under the terms and conditions of the Creative Commons Attribution (CC BY) license (https://creativecommons.org/licenses/by/4.0/).

**1. Dynamic light scattering (DLS)**

The size distribution of OFI-EVs was analyzed by DLS (Zetasizer Ultra, Malvern Panalytical, Amesbury, UK). The DLS technique analyzes the velocity distribution of particle motion caused by Brownian motion by measuring dynamic fluctuations in the intensity of scattered light. The hydrodynamic radius of the particle, or diameter considered, was calculated with the Stokes–Einstein equation. Ten microliters of purified OFI-EVs were diluted in 990 μL of filtered PBS and vortexed. To avoid aggregation of EVs, the entire volume was quickly placed into a disposable cuvette for size measurements. Three independent measurements were performed for each sample, and the analysis was processed by the software.

**2. Nanoparticles Tracking Analysis (NTA)**

NTA from Malvern (Malvern Panalytical Ltd., Malvern, Worcestershire, UK) was used for the measurement of size distribution and concentration of OFI-EVs samples in the liquid suspension. Briefly, samples were diluted with 0.2 μm-filtered PBS to obtain a recommended measurement concentration between ~128–140 particles/mL. Five videos of typical 60 s duration were taken. Data were analyzed using NanoSight NTA software version 3.2, which was optimized to first identify and then track each particle on a frame-by-frame basis with a 488 nm laser. The temperature was maintained at 25 °C. Filtered PBS (blank) was run as a negative control.

**3. Transmission Electron Microscopy (TEM) of OFI-EVs**

After isolation, OFI-EVs was resuspended in PBS and properly diluted, and then incubated for 5 min onto carbon-coated copper grids, 200 mesh at room temperature. Once absorbed on the grids, OFI-EVs was fixed with 2% glutaraldehyde in PBS for 10 min and then washed three times in Milli-Q water. Negative staining was performed with 2% phosphotungstic acid; finally, the grids were air-dried and observed by an FEI Tecnai G2 Spirit TWIN 120 kV with emission source LaB6 and mounting FEI Eagle 4k CCD camera (on the bottom) and Olympus SIS.

**4. *OFI* Total Phenolic Compounds (TPC) and Total Flavonoid Compounds (TFC)**

The extracted EVs were deconstructed using a low-temperature ultrasonic extraction solution (Ultrasonic cleaner MH-031S) to disrupt the vesicle structure, with 60% power for 2s on and 2s off, lasting for 1 min. Then, the EVs were chemically deconstructed using TritonX-100 at a volume ratio of EVs to TritonX-100 of 1:1000. The treated OFI-EVs were placed at room temperature for 30 min and dissolved into 80% methanol, and then their total phenolic and total flavonoid contents were determined.

The total phenolic content of the EVs was measured using the Folin-phenol method. Specifically, 0.3 mL of OFI-EVs was mixed with 4 mL of distilled water and 400 μL of Folin–Ciocalteu reagent [55]. After being kept in the dark for 5 min, 2 mL of $Na_2CO_3$ solution (5%) was added. The mixture was then kept in the dark for 60 min for a reaction to occur before the total phenolic content was measured using a spectrophotometer at 765 nm (Spectroquant Prove 300, Millipore).

For total flavonoid determination, 0.5 mL of the EV sample was mixed with 0.7 mL distilled water and 0.2 mL of 5% $NaNO_2$, and the mixture was shaken and placed in the dark for 6 min. Then, 0.2 mL of 10% $Al(NO_3)_3$ was added, shaken, and the mixture was again placed in the dark for 6 min. Next, 2 mL of 1 $mol{\cdot}L^{-1}$ NaOH was added, and the mixture was shaken and brought up to a total volume of 5 mL with 1.4 mL distilled water. The mixture was shaken well and placed in the dark for a further 15 min. Absorbance was measured at 500 nm.

**5. Real-Time Quantitative PCR (RT-qPCR)**

Anti-inflammatory activity was evaluated by Real-Time Quantitative PCR (RT-qPCR) according to the manufacturer's protocols. For RT-qPCR, total RNA was extracted from cells with and without OFI-EVs (5 and 20 μg/ml) stimulated with LPS (1 μg/mL) through TriFast (EuroClone, Milan, Italy), and cDNA was synthesized using Wonder RT cDNA synthesis Kit (EuroClone). Then, gene expressions of TNF-$\alpha$, IL-8, IL-6, and SOD2 were evaluated by 7900 HT fast Real-Time PCR System, (Applied Biosystem, Foster City, CA, USA) with SYBR Green PCR Master mix (EuroClone). Gene expression was quantified by the $2^{-\Delta\Delta Ct}$ method and normalized against ⊚-actin used as the internal reference gene. Results were expressed as fold changes versus control. Primers used for RT-qPCR are reported in Table S1.

Table S1: Primers used for qRT-PCR.

| Gene | Accession number | Forward (5′-3′) | Reverse (5′-3′) |
|---|---|---|---|
| SOD2 | NM_000636.4 | CTGGACAAACCTCAGCCCTA | TGATGGCTTCCAGCAACTC |
| IL-6 | NM_000600.5 | CGCCTTCGGTCCAGTTGCC | GCCAGTGCCTCTTTGCTGCTTT |
| IL-8 | NM_000584.4 | CTCTTGGCAGCCTTCCTGATTTC | TTTTCCTTGGGGTCCAGACAGAG |
| TNF-$\alpha$ | NM_000594.4 | AACATCCAACCTTCCCAAACGC | TGGTCTCCAGATTCCAGATGTCAGG |
| ACTB | NM_001101.5 | ACTCTTCCAGCCTTCCTTCC | CGTACAGGTCTTTGCGGATG |
